# Case Report: Fascioliasis Hepatica Precisely Diagnosed by Metagenomic Next-Generation Sequencing and Treated With Albendazole

**DOI:** 10.3389/fmed.2021.773145

**Published:** 2021-11-24

**Authors:** Yaling Zhang, Huan Xu, Yi Liu, Juan Kang, Hairu Chen, Zhiyi Wang, Dachuan Cai

**Affiliations:** ^1^Department of Infectious Diseases, The Second Affiliated Hospital of Chongqing Medical University, Chongqing, China; ^2^Vision Medicals Center for Infection Diseases, Guangzhou, China

**Keywords:** case report, fascioliasis hepatica, metagenomic next-generation sequencing (mNGS), precision treatment, albendazole

## Abstract

The clinical manifestations of fascioliasis hepatica in humans are unspecific. Traditional diagnosis relies on evidence of live parasites or eggs in the bile or feces. However, due to similar imaging manifestations, they are often misdiagnosed as malignant tumors. Here, we report a case of a 43-year-old woman with fever and space-occupying liver disease. Liver biopsy, parasite-specific antibody screening, and stool testing did not find any pathogens. Therefore, metagenomic next-generation sequencing (mNGS) and routine microbiological examinations were performed. Finally, *Fasciola hepatica* was only identified by mNGS. The body temperature of the patient and the eosinophil count remained normal, and the space-occupying liver lesions were significantly absorbed after more than 7 months of treatment with albendazole. The details of this case highlight the timely use of mNGS to identify parasites and judge therapeutic effects after treatment, providing important help for clinical decision-making.

## Introduction

Fascioliasis hepatica is an infection caused by a trematode of the liver*, Fasciola hepatica*, that particularly affects sheep, goats, and cattle (1). The distribution of humans infected with *F. hepatica* depends on the intermediate hosts. Due to the habitat requirements of the intermediate hosts, the distribution may be patchy (2, 3). France, the United Kingdom, the former Soviet Union, Cuba, and other countries have reported numerous cases. However, the number of patients in China is less (3), mainly scattered in the Liaoning, Jining, Heilongjiang, Jiangxi, Yunnan, Guangdong, and Guangxi provinces. More importantly, there are no cases reported in Chongqing until now. The incubation period and clinical manifestations of human fascioliasis are different and are divided into two stages. In the acute phase, the main manifestations are fever, anemia, hepatomegaly, and a significantly elevated blood eosinophils count (3–7). While in the chronic phase, it manifests as jaundice, ascites, and even spreads to the extrahepatic organs through the bloodstream (8–11). The clinical outcomes of critically ill patients are always poor. So, the confirmation of the diagnosis is necessary. In non-endemic areas, diagnosis of fascioliasis hepatica can be difficult because the disease is not often encountered and the symptoms may be confused with other hepatic or biliary disorders, such as liver abscess, liver tuberculosis, and so on. Additionally, the radiological findings in *F. hepatic* infection may present with multiple nodular lesions, subcapsular low density, and solitary nodular lesions, etc. resulting in dilemma or confusion. Presently, metagenomic next-generation sequencing (mNGS) based on high-throughput sequencing has been widely used to screen for broad-spectrum pathogens, including *Mycobacterium Tuberculosis, Toxoplasma gondii*, and other atypical pathogens. Here, we report the first case of fascioliasis hepatica identified by mNGS in Chongqing, China.

## Case Description

**Figure 1:**
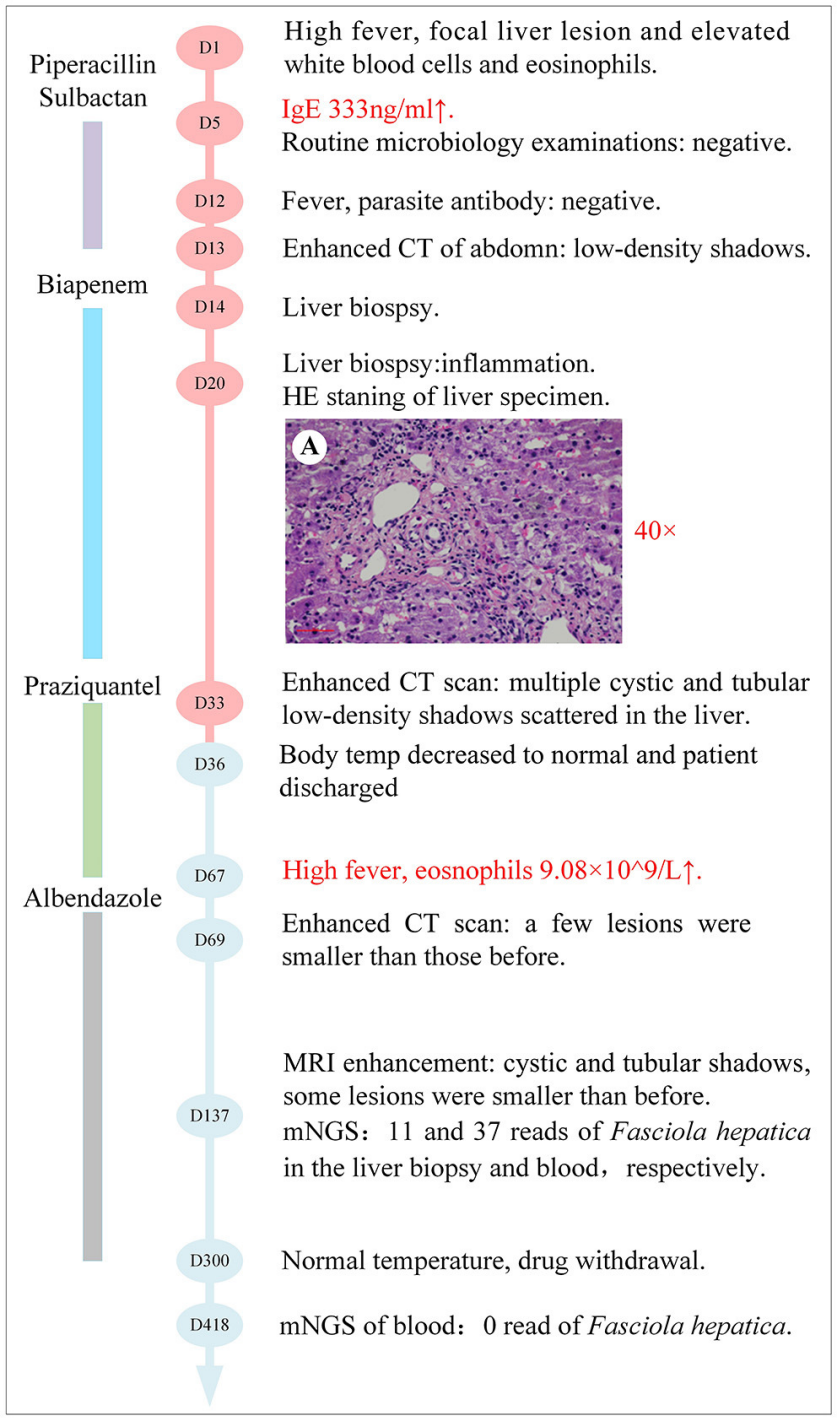
The clinical course of the patient (schematic). Hematoxylin and eosin staining of the liver specimen shows some punctate necrosis on the lobule, apoptotic bodies in the hepatic cord, fatty degeneration of hepatocytes, mild inflammation in the portal area, and eosinophil infiltration in the inflammatory necrosis area.

**Figure 2:**
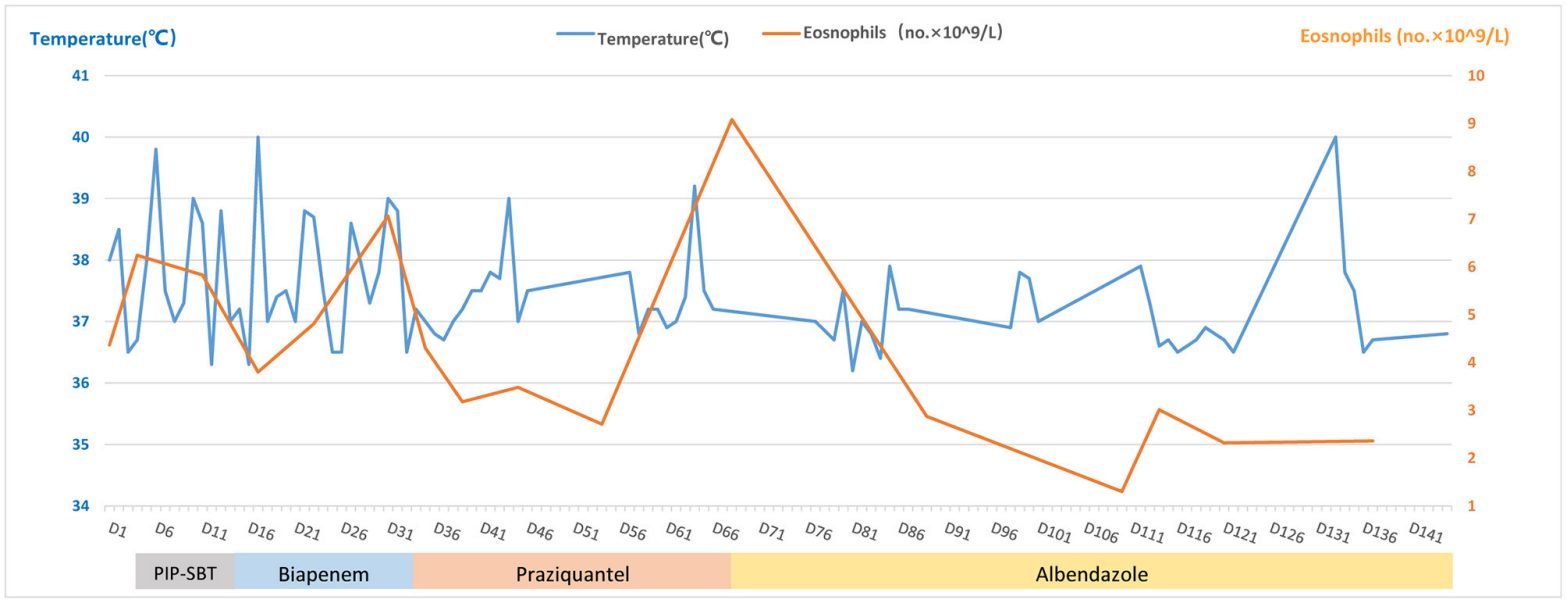
Timeline with relevant data from the episode of care; curves of body temperature and eosinophils count. The blue line shows the body temperature values. The orange line shows the eosinophil counts. Horizontal thick lines show the medications administered. PIP-SBT, Piperacillin-sulbactam.

A 43-year-old female, whose height was 160 cm and weight 46 kg [body mass index (BMI) 18.0], was admitted due to 2 months of repeated fever and space-occupying lesions in the liver on May 15, 2020 (day-1). The highest body temperature appeared at night but returned to normal in the morning, with dry cough, shortness of breath, and dull pain in the liver area. The physical examination of the whole body showed no obvious positive signs, there were no abdominal tenderness and percussion pain, and no enlarged superficial lymph nodes were palpated. So, the patient still could live and work properly. She lived in Chongqing city, whose economic situation is medium as a furniture sales staff. She visited Guangzhou province 6 months ago on business for 2 days. She denied any contact with livestock and eating wild vegetables, raw fish, shrimp, and so on, but drank tap water every day. The local hospital prescribed empirical antimicrobial therapy with azithromycin and Chinese herbal medicines; however, the treatment failed to relieve the symptoms of the patient. Thus, the patient was transferred to our hospital for further evaluation. An outline of the episodes is described in Figure 1. Chest CT scan showed no infection in the lungs, while mass-like shadows in the liver were observed on the abdominal CT scan. Routine blood parameters revealed elevated white blood cell and eosinophil count (Figure 2).

**Figure 3:**
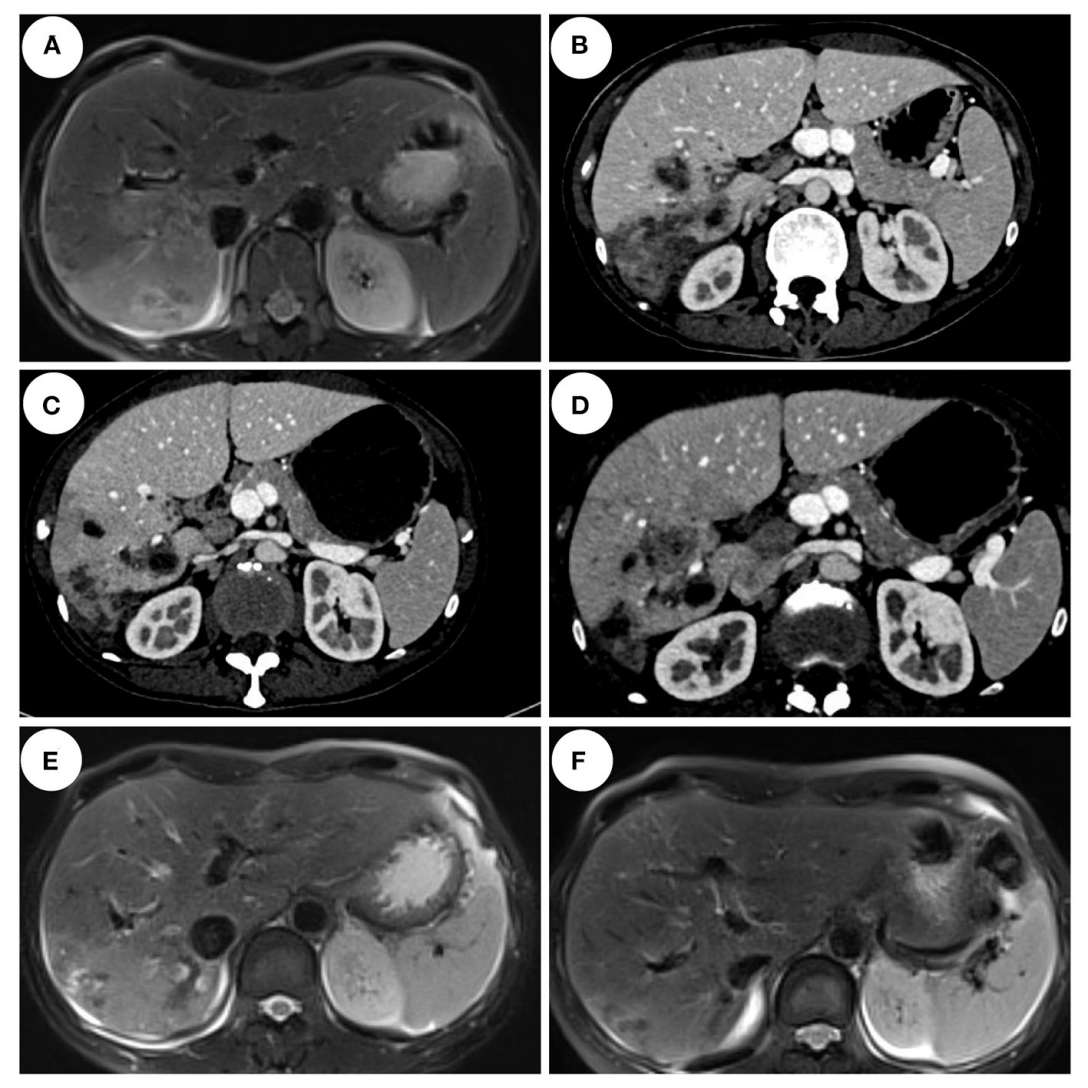
Imaging features of the patient. **(A)** Abdominal MRI enhancement showed that the right lobe of the liver scattered flakes, irregular shape, enhanced uneven enhancement on hospital day 2. **(B)** Abdominal CT enhancement showed liver multiple nodules, mass slightly low-density shadow, edge enhancement on hospital day 13. **(C)** Abdominal CT enhancement showed multiple cystic and tubular low-density shadows scattered in the liver, and some showed edge enhancement on day 33. **(D)** Abdominal CT enhancement showed multiple cystic and tubular low-density shadows scattered in the liver, some showed edge enhancement, and a few lesions were smaller than those before on follow-up day 69. **(E)** Abdominal MRI enhancement showed cystic and tubular shadows in the right lobe of the liver, with irregular shapes and edge enhancement in the enhanced part. Some lesions were smaller than before on follow-up day 137. **(F)** Abdominal MRI enhancement showed the right lobe of the liver scattered flakes, irregular shape, enhanced edge uneven enhancement, lesions significantly reduced on follow-up day 418.

Enhanced MRI of the upper abdomen showed the increased liver volume, uneven liver parenchyma signal, liver interstitial edema, and accumulated fluid in the abdominal cavity on hospital day 2 (Figure 3A). Laboratory tests showed elevated IgE (333 ng/mL), white blood cell, and eosinophil count on hospital day 5. Microbial tests, including blood cultures, stool examination, and stool precipitation, did not detect any pathogens. Eggs were not detected by direct fecal smear and fecal washing precipitation. Due to excessive pharyngeal reflex during gastroduodenal tube placement, two attempts failed to obtain the bile of the patient. The diagnosis of tuberculosis was excluded for the negative results of the chest CT scan, tuberculin skin test, tuberculosis IgG and IgM antibody screening, and T-Spot examination. Meanwhile, ovarian tumors, liver tumors, gastrointestinal tumors, and hematological tumors were also excluded temporarily as the negative results of abdominal enhanced CT and tumor marker detection such as AFP, CA-50, CEA, TAP, CA-125, CA-199, CA-242, and CA-724 and so on. The serum parasite IgG antibody, including schistosome, paragonimiasis, cysticercosis, trichinella spiralis, liver fluke, sparganosis, and hydatid IgG antibody detected by ELISA were negative on hospital day 12. Piperacillin-sulbactam was used as an antibacterial treatment for 10 days from hospital days 4–13.

However, the body temperature of the patient fluctuated several times, and the eosinophil count was not significantly reduced (Figure 2). So, an enhanced CT of the lower abdomen was performed on hospital day 13, which showed multiple shadows of abnormal density in the liver and a small amount of effusion, suggesting the possibility of infectious and neoplastic lesions (Figure 3B). Because of the high fever, biapenem was adjusted to be used to cover drug-resistant bacteria for more than 2 weeks from hospital day-14 to day-32.

To further confirm the diagnosis, an ultrasound-guided percutaneous liver biopsy was conducted on hospital day-14. The hepatic histopathology reported mild inflammation in the portal area and eosinophil infiltration in the inflammatory necrotic area, with no evidence of malignancy, liver abscess, and parasitic infection on hospital day 20 (Figure 1).

As treatment with antibiotics was ineffective (long-term fever, significantly elevated eosinophils count, intrahepatic lesions), the enhanced MRI of the upper abdominal was re-examined on hospital day 33, which indicated no absorption of intrahepatic lesions (Figure 3C). Meanwhile, combined with Chongqing epidemiological studies of *Clonorchis sinensis*, parasite infection was suspected and praziquantel was used as treatment. The body temperature decreased, and the patient was discharged on day 36.

During the period of hospital days 33 and 67, the patient still had repeated fever with a maximum temperature of 39°C (Figure 2). An enhanced MRI of the upper abdominal showed that the liver shadow had less absorption (Figure 3D). So, albendazole was adjusted as the treatment on the follow-up day. Albendazole was orally taken 0.8 g once a day for half a month. As the body temperature of the patient decreased, the dose of albendazole was decreased to 0.6 g for half a month after drug withdraw. On the follow-up day, the dose of albendazole was not adjusted and the patient was prescribed to take albendazole according to taking half a month and stopping for half a month. Firstly, the peak temperature of the body decreased significantly. Secondly, the eosinophil count decreased and ultimately returned to normal. However, there was still a high fever up to 40°C (Figure 2), occasionally. Meanwhile, the enhanced MRI of the upper abdominal reexamined on follow-up day 137 showed that there was no significant absorption of the lesions compared with the previous (Figure 3E). So far, after a total of 106 days of empirical anti-parasitic treatment, the body temperature and eosinophil count decreased significantly, but the imaging manifestations did not improve significantly.

**Figure 4:**
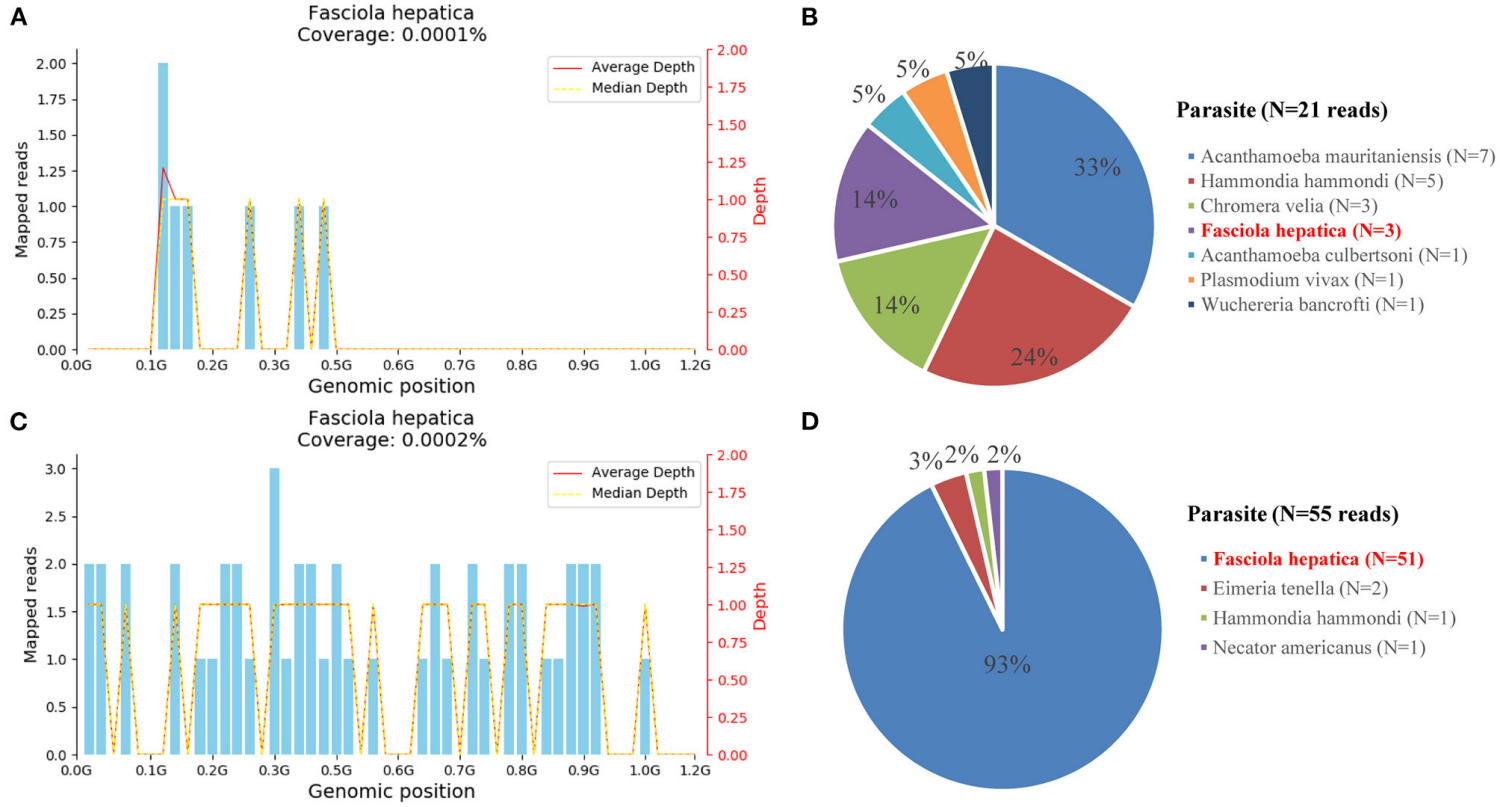
Diagnosis of Fasciola hepatica infection using metagenomic next-generation sequencing (mNGS). **(A)** The reads mapped to the Fasciola hepatica genome with coverage of 0.0001% in the liver tissue. **(B)** The distribution of species-specific reads aligning to the parasite in the liver tissue. **(C)** The reads mapped to the Fasciola hepatica genome with coverage of 0.0002% in the blood. **(D)** The distribution of species-specific reads aligning to the parasite in the blood.

To identify the pathogen, liver tissue collected on hospital day 14 and peripheral blood on follow-up day 137 were sent for mNGS with the written consent of the patient. The total reads of the liver tissue and blood were 6.93 and 30,379 Mb, respectively. Meanwhile, the reads mapped to microbes in the liver tissue and blood were 934,425, and 29,009. The number of species-specific reads aligning to the Fasciola hepatica genome in the liver biopsy and blood was 3 and 51, respectively (Figure 4). Finally, the disease was diagnosed with fascioliasis hepatica with triclabendazole as the first-line drug (4). However, for the sake of the unavailability of triclabendazole under the epidemic of COVID-19 in China and other countries, and the partial effectiveness of albendazole on the fascioliasis hepatica which significantly decreased the body temperature and eosinophil count of the patient, albendazole was used with a prolonged treatment time following the previous way.

Eventually, after receiving treatment of albendazole for more than 7 months, the body temperature and eosinophil count of the patient normalized with no obvious clinical manifestations. So, albendazole was discontinued on day-300. After more than 3 months after drug withdrawal on follow-up day 148, the enhanced MRI of the upper abdominal showed that the intrahepatic low-density shadow was smaller (Figure 3F), and the blood mNGS was negative (Table 1), suggesting the albendazole treatment was effective.

**Table 1:** Results of metagenomic next-generation sequencing (mNGS) in the liver tissue and blood.

**Time of mNGS detection**	**Sample type**	**Species-specific reads of *Fasciola hepatica***	**Other microbes (Species-specific reads)**
Day 138	Liver tissue (collected on day 14)	3	*Methyloversatilis Discipulorum* (389, 801)
	Peripheral blood	51	*Klebsiella variicola* (72) *Torque teno virus* (5)
Day 418	peripheral blood	0	-

## Discussion

To our knowledge, this is the first case report of fascioliasis hepatica confirmed by mNGS, which played an important role in this case. Fascioliasis hepatica is often sporadic and has not been reported previously in Chongqing, China. The source of infection is contaminated water containing larva and aquatic plants and is easily ignored by clinicians in medical history collection. The diagnosis of fascioliasis hepatica may be difficult because of the lack of a clear epidemiological history, wide spectrum of differential diagnosis, and low incidence (3). The abnormal laboratory and radiological findings in *F. hepatica* infection may represent hepatitis, liver abscess, malignancy, and infection with parasites such as *Ascariasis* and *Clonorchis sinensis*. More recently, triclabendazole is the drug of choice for its effectiveness against both adult and immature worms (7, 12, 13). As for albendazole, it is partially effective against fascioliasis hepatica. In this case, triclabendazole was not obtained for treatment due to the epidemic of the coronavirus disease 2019 (COVID-19). After treatment with albendazole, the body temperature and eosinophil count of the patient decreased significantly. Albendazole is considered partially effective. In this particular case, both the MRI and CT of the upper abdomen showed abnormal uneven enhancement density shadows that are more likely to be considered as infectious lesions, but tumor lesions cannot be excluded. This may be related to the atypical imaging of fascioliasis hepatica. Such lesions pose a diagnostic dilemma or confusion between malignancy and infection (3). Also, there was no finding of parasites or eggs in the hepatic tissue, which was destroyed when making sliced specimens. Currently, diagnosis is confirmed only by demonstrating live parasites or eggs in the bile or feces, through Fecal Sedimentation, Modified Kato-Katz thick smear method, and Mercury-aldehyde iodine concentration method, but with a relatively low positive rate (4, 5). An examination of the precipitation or centrifugation of the duodenal fluid has a relatively high positive rate (14). Also, demonstrating live parasites or eggs by laparotomy, laparoscopy biopsy, and other histopathologic examination is less used in the clinic for the larger trauma.

However, there is no egg in the stool of the patient in the acute stage. The acute stage of fascioliasis hepatica begins with the slow migration of *F. hepatica* through the liver parenchyma; the mature flukes digest and consume hepatocytes, dig tunnels and caves, and reside in the liver for months, causing hepatitis, chills, fever, right upper abdominal pain, abdominal distension, and loss of appetite (4, 5, 7, 9, 15). While in the chronic stage, the worm moves to the bile duct, and the inner wall is digested, causing bile duct dilatation, cholangitis, cholelithiasis, and liver pain, jaundice, and other discomforts (9–11). But the clinical manifestations are atypical and difficult to diagnose. Therefore, it requires more diagnostic tools to assist in the diagnosis. In the acute stage, serum immunological examination, such as ELISA, indirect fluorescent antibody test (IFA), indirect hemagglutination test (IHA), and counter-current immune-electrophoresis (CIE), can be used as identification tools (16–19). But the serological tests may have similar results with other schistosome infections. Therefore, it is crucial to find early etiological evidence.

Metagenomic next-generation sequencing, with fast detection speed, broad coverage, and high accuracy, is a new type of less biased detection method (20–22), which can detect thousands to millions of nucleotides fragments at once (20, 21, 23, 24). The suspected pathogen can be detected by the mNGS, through the flow of sequencing, data cleaning, error detection, and database comparative analysis. At present, mNGS has been widely used in clinics, including, respiratory tract infection (25), central nervous system infection (26), joint infection (27, 28), hepatic tuberculosis (29), etc. Metagenomic next-generation sequencing has many advantages in identifying hard-to-culture, atypical, parasite, and rare pathogens, such as *Mycobacterium Tuberculosis* and *Toxoplasma gondii*. On the other side, it also has shortcomings such as its high cost, complex process, specimen contamination, etc. *Fasciola hepatica* was detected both in the blood and liver tissues of this case, with corresponding treatment, the clinical symptoms and imaging manifestations were recovered. The peripheral blood was sent for mNGS again after drug withdrawal, and no reads of *Fasciola hepatica* were detected. It proved the effectiveness and specificity of mNGS for the detection of parasites. We believe that the application of mNGS in pathogen identification will guide the clinical diagnosis and treatment, and then greatly improve the accuracy and benefit patients.

## Data Availability Statement

The original contributions presented in the study are included in the article, further inquiries can be directed to the corresponding authors.

## Ethics Statement

Written informed consent was obtained from the patient for the publication of any potentially identifiable images or data included in this article.

## Author Contributions

ZW and HX contributed to the conception and design of the study. YZ and HX collected the data and wrote the manuscript. HX, YL, JK, and HC analyzed and interpreted the patient data. ZW and DC reviewed the manuscript. All authors read through and approved the final manuscript.

## Funding

This work was supported by the Natural Science Foundation of Chongqing CSTC (No. cstc2020jcyj-msxmX0015).

## Conflict of Interest

The authors declare that the research was conducted in the absence of any commercial or financial relationships that could be construed as a potential conflict of interest.

## Publisher's Note

All claims expressed in this article are solely those of the authors and do not necessarily represent those of their affiliated organizations, or those of the publisher, the editors and the reviewers. Any product that may be evaluated in this article, or claim that may be made by its manufacturer, is not guaranteed or endorsed by the publisher.
